# Artificial intelligence in education: effects on motor learning, motivation, and student engagement from an educational psychology perspective

**DOI:** 10.3389/fpsyg.2026.1816494

**Published:** 2026-08-28

**Authors:** Amin Daly, Sofiene Mnedla, Mohamed Souhaiel Chelly

**Affiliations:** 1 Research Laboratory (LR23JS01) “Sport Performance, Health & Society”, University of Manouba, Tunis, Tunisia; 2 Higher Institute of Sport and Physical Education of Ksar Said, University of Manouba, Tunis, Tunisia; 3 Higher Institute of Applied Studies in Humanities, Tunis University, Zaghouan, Tunisia

**Keywords:** artificial intelligence, educational psychology, intrinsic motivation, motor learning, secondary education, student engagement

## Abstract

The rapid expansion of artificial intelligence (AI) in education has renewed debates about its pedagogical value, particularly in relation to learner-centered approaches and motivational processes. While a growing body of research has examined AI-supported learning in cognitively oriented subjects, empirical evidence remains scarce in practice-based disciplines such as physical education (PE), especially within secondary school contexts in developing countries. This study investigated the effects of integrating AI-supported instructional tools into PE lessons on students' motor learning, intrinsic motivation, and engagement in Tunisian secondary schools. A quasi-experimental pre-test/post-test design involving two intact classes was employed because individual random assignment was not feasible in the school setting (*N* = 56; age range 15–17 years, *M* = 16.4, SD = 0.9), including an experimental group receiving AI-supported instruction and a control group following traditional teaching methods over an 8-week intervention period. Motor learning was assessed using the TGMD-3, intrinsic motivation through the Intrinsic Motivation Inventory, and engagement via a multidimensional student engagement scale. Results revealed statistically significant and educationally meaningful improvements in motor skill acquisition, intrinsic motivation, and engagement among students exposed to AI-supported instruction compared with their peers in the control group. Effect sizes indicated strong practical relevance, suggesting that AI-supported visual feedback based on predefined movement indicators was interpreted and contextualized by the teacher to support motor skill acquisition. These findings suggest that AI-supported instruction may enhance motor learning, intrinsic motivation, and student engagement when integrated into teacher-mediated physical education. Given the quasi-experimental design involving intact classes, these findings should be interpreted as evidence of association rather than definitive causal effects. These findings contribute context-sensitive empirical evidence to the field of educational psychology by highlighting the potential of human-centered AI integration in secondary school physical education within a developing country context. The study underscores the importance of pedagogical mediation, learner autonomy, and ethical awareness in the design and implementation of AI-supported learning environments.

## 1 Introduction

### 1.1 Artificial intelligence and contemporary educational change

Artificial intelligence (AI) has increasingly shaped contemporary educational debates over the last decade, not only as a technological innovation but also as a catalyst for rethinking pedagogical practices, assessment processes, and learner support systems (Chen et al., 2020). Recent developments in machine learning and learning analytics have contributed to positioning AI at the center of discussions on educational transformation, influencing how knowledge is accessed, mediated, and constructed within learning environments (Luckin and Holmes, 2016). From this perspective, AI is no longer viewed merely as a set of automated tools but as part of a broader socio-technical ecosystem that reshapes educational practices and institutional logics (Williamson and Eynon, 2020).

Several international frameworks emphasize that the educational value of AI depends less on technological sophistication than on the pedagogical models within which it is embedded. In this regard, international guidance highlights the importance of aligning AI applications with ethical principles and educational goals, particularly those related to equity, inclusion, and learner empowerment (Miao and Holmes, 2021). Complementarily, policy-oriented analyses conducted by the OECD stress that AI should be integrated in ways that support teachers' professional agency and enhance learning opportunities rather than reinforcing technocentric reforms (Bo, 2025).

Empirical reviews of AI in education research converge on the idea that learning gains associated with AI-supported environments emerge primarily when such systems enhance feedback quality, personalization, and opportunities for self-regulation (Zawacki-Richter et al., 2019). However, these effects remain contingent upon instructional design and teacher mediation, as underlined by studies showing that poorly integrated AI tools yield limited pedagogical benefits (Roll and Wylie, 2016). From an educational psychology perspective, this observation reinforces the need to situate AI within coherent pedagogical frameworks that account for learners' cognitive and motivational processes (Holmes et al., 2019).

### 1.2 Gaps in the literature: practice-based disciplines

Despite the rapid expansion of empirical research on artificial intelligence in education, existing studies remain heavily concentrated in cognitively oriented subjects such as mathematics, science, and language learning, where learning outcomes can be more easily operationalized and measured (Aleven et al., 2016). While this body of work has contributed valuable insights into intelligent tutoring systems and adaptive learning platforms, it offers limited guidance for understanding AI integration in learning contexts characterized by embodied and situated practices.

Physical education represents a particularly underexplored domain in this regard. Learning in PE is grounded in motor experiences, bodily coordination, and social interaction, making learning processes less visible and more difficult to capture through conventional assessment tools (Kirk, 2009). Although previous research on digital technologies in PE has shown that tools such as video-based feedback can support reflective learning and skill acquisition when pedagogically integrated (Goodyear et al., 2014), most of these studies focus on digital mediation rather than on AI-supported systems. As a result, the specific contribution of AI-based feedback to learning processes in secondary school PE contexts remains insufficiently documented (Koekoek and Van Hilvoorde, 2019). Recent systematic reviews and meta-analyses have reported generally positive effects of AI-supported educational interventions on academic achievement and learner engagement, although effect sizes vary substantially depending on instructional design, subject domain, and the degree of teacher mediation (Zawacki-Richter et al., 2019; Crompton and Burke, 2023). Nevertheless, evidence from practice-based disciplines such as physical education remains limited, particularly in authentic secondary-school settings and developing-country contexts. Furthermore, few studies have simultaneously examined motor learning, intrinsic motivation, and student engagement within a single theoretically grounded framework. These methodological gaps provide the rationale for the present study.

Furthermore, systematic reviews indicate that the presence of technology alone does not guarantee enhanced learning outcomes in physical education. Without coherent pedagogical framing, digital tools may become peripheral or even disruptive within lesson dynamics, a concern highlighted in critical analyses of technology use in PE (Ennis, 2017). These observations point to the need for empirically grounded research examining AI-supported instruction under authentic school conditions rather than in laboratory or elite sport settings.

### 1.3 Learning in physical education: motor, motivational, and social dimensions

Learning in physical education is inherently multidimensional, encompassing motor, cognitive, affective, and social dimensions that interact dynamically throughout the learning process (Ennis, 2017). From a motor learning perspective, skill acquisition is conceptualized as a non-linear and context-dependent process shaped by task constraints, practice conditions, and informational feedback (Newell, 1986). Within this framework, learning involves continuous adaptation and refinement through exploration, error, and feedback rather than the simple reproduction of prescribed movement patterns (Schmidt et al., 2018).

Feedback occupies a central position in motor learning, as it directs learners' attention toward task-relevant information and supports the regulation of movement execution. Educational psychology research has demonstrated that feedback effectiveness depends on its timing, frequency, and informational content (Hattie and Timperley, 2007). However, in school-based PE contexts, structural constraints such as large class sizes and limited instructional time often restrict teachers' capacity to provide individualized and immediate feedback to all learners (Kirk, 2009). This can result in generalized or delayed feedback, potentially limiting opportunities for optimal motor learning.

Beyond motor dimensions, motivational and social factors play a decisive role in shaping students' engagement in physical education. Empirical research indicates that student engagement in PE is closely linked to motivational orientations and classroom climate (Fredricks et al., 2004). Self-determination theory further highlights that intrinsic motivation is fostered when learning environments support learners' needs for autonomy, competence, and relatedness (Ryan and Deci, 2000). In this sense, pedagogical approaches and technological tools that make individual progress visible and support self-assessment may influence not only performance outcomes but also students' willingness to engage meaningfully in learning activities (Reeve, 2012).

### 1.4 Artificial intelligence as a pedagogical mediator in physical education

From a pedagogical standpoint, artificial intelligence can be conceptualized as a mediating artifact within broader learning systems rather than as an autonomous instructional agent. This perspective aligns with socio-cultural approaches to learning, which emphasize that cognitive processes are distributed across individuals, tools, and environments (Salomon, 1997). In educational settings, technological tools shape how learners perceive tasks, access information, and regulate their activity, thereby influencing learning trajectories in indirect yet meaningful ways (Engeström, 2001).

Within physical education, AI-supported tools such as semi-automated video analysis and movement recognition systems can potentially render motor learning processes more visible. By providing visual and quantitative representations of performance, these tools may complement teachers' observational feedback and support learners' reflective practice (Baca and Kornfeind, 2012). Empirical work in sports science further indicates that wearable sensors and computer vision systems can enhance the precision of movement analysis when aligned with pedagogical goals (Camomilla et al., 2018).

However, the pedagogical value of such technologies remains contingent upon how feedback is framed and interpreted within instructional contexts. Studies in educational technology and learning analytics caution that data-driven feedback can be ineffective or even counterproductive if it is disconnected from pedagogical intentions or experienced by learners as controlling (Selwyn, 2019). This underscores the importance of teacher mediation in transforming AI-generated information into meaningful learning cues, consistent with human-centered AI frameworks that emphasize transparency and pedagogical alignment (Holmes et al., 2022).

### 1.5 The Tunisian secondary school context

Research on artificial intelligence in education has largely focused on technologically advanced contexts, often overlooking the realities of public schooling in developing countries (Trucano and Trucano, 2016). In Tunisia, secondary education operates under structural constraints that include large class sizes, limited material resources, and strong curricular pressures, which shape both teaching practices and students' learning experiences. These contextual factors are particularly salient in physical education, a compulsory subject that nonetheless faces challenges related to student disengagement and limited instructional differentiation (Kirk, 2009; Trucano and Trucano, 2016).

Recent research in physical education highlights substantial variations in student engagement and participation patterns, indicating that pedagogical approaches and classroom climate play a critical role in shaping learning experiences across secondary school contexts (Fredricks et al., 2004; Reeve, 2012). At the policy level, national strategies increasingly emphasize digital transformation in education, echoing broader international agendas. However, the integration of advanced technologies such as AI remains uneven, and empirical evidence regarding their pedagogical impact in PE is still scarce. This situation calls for context-sensitive investigations that examine not only technological feasibility but also pedagogical relevance within the Tunisian schooling system.

These contextual constraints also influenced the methodological choices adopted in the present study, including the use of intact classes and an intervention designed to be feasible within the realities of public secondary schools. Accordingly, the intervention relied on a low-cost AI-supported video feedback approach that could be implemented using resources typically available in Tunisian public secondary schools. Outcome measures were selected because they assess complementary dimensions of learning—motor performance, intrinsic motivation, and student engagement—while remaining feasible to administer within regular physical education lessons. These methodological choices were intended to maximize ecological validity while respecting the practical constraints of the educational setting.

### 1.6 Research problem and objectives

Building on the gaps identified in the literature and the specificities of the Tunisian context, the present study addresses the following research problem: to what extent can the integration of AI-supported instructional tools enhance motor learning, intrinsic motivation, and student engagement in secondary school physical education? This problem is grounded in theoretical assumptions drawn from motor learning research, which highlights the role of feedback and practice conditions in skill acquisition (Guadagnoli and Lee, 2004), as well as from motivational theories emphasizing the importance of perceived competence and autonomy in sustaining engagement (Ryan and Deci, 2000).

In line with these theoretical foundations, the study pursues four interrelated objectives. First, it seeks to examine the effects of AI-supported instruction on motor learning outcomes in PE, building on established models of motor skill acquisition (Schmidt et al., 2018). Second, it aims to analyze changes in students' intrinsic motivation following AI integration, with reference to self-determination theory (Deci and Ryan, 2008). Third, the study assesses the impact of AI-supported instruction on student engagement during PE lessons, drawing on multidimensional conceptualizations of engagement (Fredricks et al., 2004). Finally, it intends to contribute empirically grounded evidence from a developing country context to the international literature on AI in education (Zawacki-Richter et al., 2019; Crompton and Burke, 2023).

Because intact classes were used in this quasi-experimental study, the findings should be interpreted as evidence of associations between AI-supported instruction and educational outcomes rather than as definitive causal effects. This methodological consideration guided both the study design and the interpretation of the results. Based on the theoretical framework and previous empirical evidence, we hypothesized that students receiving AI-supported instruction would demonstrate greater improvements in (H1) motor learning, (H2) intrinsic motivation, and (H3) student engagement than students receiving traditional instruction. Given the quasi-experimental design, these hypotheses were formulated to examine expected associations rather than definitive causal relationships.

### 1.7 Significance of the study

By focusing on a practice-based discipline within a secondary school context in Tunisia, this study responds to calls for more contextually grounded and theoretically informed research on artificial intelligence in education. Rather than advancing a deterministic vision of technological innovation, the study adopts a human-centered perspective in which AI is examined as a pedagogical support tool embedded within existing teaching practices (Floridi et al., 2018). Such an approach aligns with critical perspectives that emphasize the relational, ethical, and embodied dimensions of educational practice, particularly in subjects such as physical education (Williamson and Eynon, 2020).

The findings are expected to inform researchers, educators, and policymakers seeking to understand how AI can be integrated into physical education in ways that enhance learning while preserving the human and social foundations of schooling. In this respect, the study contributes to ongoing international debates on responsible and pedagogically grounded AI integration in education, as reflected in recent policy frameworks and ethical guidelines (Miao and Holmes, 2021; Bo, 2025).

To our knowledge, few quasi-experimental studies have examined AI-supported instruction in secondary school physical education within developing-country contexts. The present study contributes context-specific evidence from Tunisia and extends the literature by integrating motor learning theory, self-determination theory, and distributed cognition within a single educational framework.

## 2 Theoretical framework

### 2.1 Artificial intelligence in education: from automation to pedagogical mediation

Early approaches to artificial intelligence in education were largely oriented toward automation, with a primary focus on content delivery, automated assessment, and the replacement of routine instructional tasks. Such technocentric visions often assumed that the integration of intelligent systems would, in itself, lead to improved learning outcomes. However, empirical research has progressively challenged this assumption, showing that technological automation does not automatically translate into pedagogical effectiveness (Roll and Wylie, 2016).

More recent perspectives conceptualize AI as a pedagogical mediator embedded within broader learning systems rather than as an autonomous instructional agent. In this learning-oriented paradigm, AI-supported environments contribute to education by enhancing feedback quality, supporting personalization, and facilitating learners' self-regulation processes (Luckin and Holmes, 2016). Large-scale reviews further indicate that the effectiveness of AI-supported learning environments depends strongly on instructional design choices and the extent to which teachers remain actively involved in mediating technological feedback (Zawacki-Richter et al., 2019).

From an educational psychology standpoint, this shift reflects a broader move away from deterministic views of technology toward socio-constructivist and human-centered approaches to learning. Human-centered AI frameworks emphasize transparency, interpretability, and teacher agency as central principles guiding the responsible integration of AI in educational settings (Floridi et al., 2018). In line with this perspective, Holmes and colleagues argue that AI should be designed to augment teachers' professional judgment rather than to substitute pedagogical decision-making processes (Holmes et al., 2022).

### 2.2 Motor learning theory and feedback in physical education

Motor learning theory provides a foundational framework for understanding how AI-supported feedback may influence learning processes in physical education. Classical and contemporary models describe motor skill acquisition as a non-linear process shaped by task constraints, practice conditions, and informational feedback (Newell, 1986). Learning is thus conceptualized as an adaptive process involving continuous interaction between the learner and the environment rather than as the linear accumulation of correct responses (Schmidt et al., 2018).

Feedback plays a central role in directing learners' attention toward task-relevant information and facilitating movement refinement. Educational research has demonstrated that feedback effectiveness depends on its timing, frequency, and informational content, with overly frequent or prescriptive feedback potentially undermining learners' autonomy and long-term retention (Hattie and Timperley, 2007). Complementarily, motivational perspectives on motor learning suggest that feedback that supports perceived competence and autonomy can enhance engagement and persistence in practice tasks (Wulf and Lewthwaite, 2016).

In school-based physical education contexts, teachers often face structural constraints, including large class sizes and heterogeneous ability levels, which limit opportunities for individualized feedback (Kirk, 2009). Within this context, AI-supported tools such as automated video analysis and movement recognition systems may offer complementary sources of performance-related information that extend teachers' observational capacity (Baca and Kornfeind, 2012). When aligned with pedagogical goals, such tools can support reflective practice and more differentiated feedback processes (Camomilla et al., 2018).

### 2.3 Motivation, engagement, and self-determination theory

Motivation and engagement are widely recognized as central mediators of learning outcomes in physical education, particularly during adolescence, a developmental period marked by increasing heterogeneity in students' attitudes toward school-based physical activity (Fredricks et al., 2004). From an educational psychology perspective, engagement is conceptualized as a multidimensional construct encompassing behavioral, cognitive, and affective components that interact dynamically throughout the learning process (Henrie et al., 2015).

Self-determination theory provides a robust theoretical framework for understanding how learning environments shape motivational orientations. According to this theory, intrinsic motivation is fostered when learners' basic psychological needs for autonomy, competence, and relatedness are supported (Ryan and Deci, 2000). Empirical research further suggests that instructional environments emphasizing meaningful choice and constructive feedback tend to promote higher levels of sustained engagement and persistence in learning activities (Deci and Ryan, 2008).

Within technology-enhanced learning environments, AI-supported feedback has been proposed as a pedagogical resource that may support learners' motivation by making performance criteria more transparent and progress more visible, particularly when interpreted and mediated by teachers.

Studies in educational technology indicate that such feedback can enhance perceived competence and self-efficacy when learners are guided in interpreting performance information (Sun and Rueda, 2012). However, motivational benefits are not inherent to technology use; they depend on whether learners perceive AI-supported systems as autonomy-supportive rather than controlling, a concern highlighted in critical analyses of educational technologies (Selwyn, 2019).

### 2.4 Distributed cognition and the teacher's role

From a distributed cognition perspective, learning is understood as an emergent property of interactions among learners, teachers, tools, and environments (Salomon, 1997). Within this framework, AI-supported technologies constitute components of a broader cognitive system that shapes how information is processed, interpreted, and acted upon. This perspective challenges individualistic accounts of learning by emphasizing the mediating role of artifacts and social relations in cognitive activity (Engeström, 2001).

In educational contexts, the pedagogical value of AI-generated data depends critically on teachers' capacity to interpret and contextualize information in light of learners' needs and curricular objectives. Research in learning analytics demonstrates that data-driven feedback only becomes educationally meaningful when educators actively engage in sense-making processes and integrate insights into instructional decision-making (Pardo and Siemens, 2014). Complementary studies further show that teachers' professional judgment plays a decisive role in determining whether analytics-informed interventions support or hinder learning (Wise and Shaffer, 2015).

In physical education, teachers' interpretive role is particularly salient given the embodied and situated nature of learning processes. AI-supported feedback must therefore be translated into actionable pedagogical guidance that respects learners' developmental trajectories and emotional experiences. Human-centered AI approaches explicitly recognize this dynamic, advocating for systems designed to support ather than supplant teacher agency and professional expertise (Holmes et al., 2022).

### 2.5 Conceptual model of the study

Drawing on motor learning theory, self-determination theory, and distributed cognition frameworks, the present study adopts a conceptual model in which AI-supported instructional tools influence learning outcomes through their effects on feedback quality, intrinsic motivation, student engagement, and learners' self-regulatory processes. In this model, AI-supported tools function as mediating artifacts that enhance the visibility and interpretability of performance-related information, thereby supporting reflective practice and learner autonomy.

Specifically, AI-supported tools are assumed to contribute to enhanced feedback quality by providing timely, task-relevant, and individualized performance cues. Enhanced feedback quality is, in turn, expected to support learners' perceived competence and engagement in learning activities, consistent with motivational principles articulated in self-determination theory (Ryan and Deci, 2000) and feedback research in educational psychology (Hattie and Timperley, 2007). Through these mediating processes, improvements in motor learning outcomes are anticipated, in line with motor learning frameworks emphasizing the role of feedback and practice conditions in skill acquisition (Schmidt et al., 2018).

### 2.6 Research hypotheses

Grounded in the theoretical framework and prior empirical findings, the study tests the following hypotheses:

- H1: Students receiving AI-supported instruction will demonstrate greater improvements in motor learning than those receiving traditional instruction (Guadagnoli and Lee, 2004; Schmidt et al., 2018).
- H2: AI-supported instruction will lead to higher levels of intrinsic motivation compared to traditional PE instruction (Ryan and Deci, 2000; Sun and Rueda, 2012).
- H3: Student engagement will increase significantly in the AI-supported group relative to the control group (Fredricks et al., 2004; Henrie et al., 2015).

## 3 Empirical literature review

### 3.1 Empirical evidence on artificial intelligence in education

Over the past decade, empirical research on artificial intelligence in education has expanded substantially, with a gradual shift from descriptive accounts of technological affordances toward more outcome-oriented evaluations of learning processes. Large-scale reviews indicate that AI-supported learning environments can yield positive effects on academic achievement when they are designed to enhance formative feedback, personalization, and learners' self-regulation capacities (Zawacki-Richter et al., 2019). These effects, however, are not uniform across contexts and learner populations, underscoring the importance of pedagogical design and contextual sensitivity.

Studies focusing on intelligent tutoring systems and adaptive learning platforms report moderate to large effect sizes when compared with traditional instructional approaches, particularly in cognitively oriented domains (Aleven et al., 2016). Nevertheless, meta-analytic syntheses caution that such gains depend heavily on how AI-supported systems are embedded within instructional practices and on learners' capacity to interpret and act upon system-generated feedback (Ouyang et al., 2022). In this sense, AI effectiveness is less a function of automation *per se* than of pedagogical integration and teacher mediation (Roll and Wylie, 2016).

From a learning analytics perspective, empirical investigations further demonstrate that data-driven feedback enhances learning only when educators actively engage with analytics outputs and incorporate insights into pedagogical decision-making processes (Pardo and Siemens, 2014). This finding reinforces human-centered perspectives on AI in education, which emphasize the centrality of teacher agency in shaping the educational value of technological innovations (Holmes et al., 2022).

### 3.2 Digital and AI-supported technologies in physical education

Empirical research on technology use in physical education predates the recent focus on artificial intelligence and provides an important foundation for understanding current developments. Studies examining video-based feedback in PE contexts report improvements in learners' movement awareness, self-assessment skills, and engagement when such tools are integrated within coherent pedagogical designs (Goodyear et al., 2014). These findings suggest that visual mediation can support reflective practice and facilitate learners' understanding of movement quality.

Systematic reviews of digital technology use in PE further indicate that technological tools can positively influence learning outcomes when they are aligned with curricular objectives and embedded within lesson structures (Koekoek and Van Hilvoorde, 2019). However, these reviews also highlight considerable variability in outcomes, with some studies reporting limited or mixed effects when technologies are introduced without clear pedagogical intent (Ennis, 2017). This variability underscores the importance of instructional design in mediating the educational impact of digital tools.

Empirical evidence specifically addressing AI-supported technologies in PE remains limited, particularly at the secondary school level. Existing studies are often conducted in higher education, elite sport training, or laboratory contexts rather than in authentic school environments (Baca and Kornfeind, 2006). Consequently, there is a need for school-based research that examines how AI-supported feedback systems function under everyday instructional conditions and within diverse learner populations.

### 3.3 AI-supported feedback and motor skill acquisition

Motor learning research provides a strong theoretical and empirical foundation for understanding the potential contribution of AI-supported feedback to skill acquisition. Empirical studies in sports science contexts indicate that augmented feedback, particularly when delivered through visual or kinematic representations, can facilitate learners' understanding of movement mechanics and accelerate performance improvements (Baca and Kornfeind, 2012). These effects are most pronounced when learners are supported in interpreting feedback information and relating it to task-relevant cues.

Investigations into automated movement analysis systems suggest that AI-supported feedback can enhance performance accuracy by making critical aspects of movement execution more salient (Camomilla et al., 2018). However, motor learning theory cautions that overly detailed or prescriptive feedback may constrain learners' exploratory behavior and reduce opportunities for adaptive learning (Wulf and Lewthwaite, 2016). This tension highlights the need for pedagogical mediation in determining the optimal level and form of feedback.

In school-based PE contexts, where learners' abilities and motivational profiles vary widely, AI-supported feedback may be particularly valuable when it complements teachers' observational feedback rather than replacing it. This hybrid approach aligns with findings from learning analytics research, which emphasize the importance of reflective dialogue and teacher-guided interpretation of performance data (Wise and Shaffer, 2015).

### 3.4 Motivation and engagement in AI-enhanced learning environments

Motivation and engagement have emerged as central outcome variables in empirical studies of technology-enhanced learning. Research grounded in educational psychology consistently shows that learners' motivational orientations condition the extent to which they benefit from instructional interventions, particularly in contexts that require sustained effort and practice (Ryan and Deci, 2000). Within AI-enhanced learning environments, empirical evidence suggests that feedback-rich systems can support students' perceived competence and foster more positive motivational trajectories when they are aligned with pedagogical intentions (Sun and Rueda, 2012).

Studies examining student engagement in digital and AI-supported learning environments further indicate that technologies can promote behavioral, cognitive, and affective engagement when they provide clear goals, timely feedback, and opportunities for self-regulation (Schindler et al., 2017). However, engagement gains are not inherent to the use of technology. Critical analyses highlight that learners may disengage when technological systems are perceived as surveillance tools or mechanisms of external control rather than as supports for learning (Selwyn, 2019). This finding underscores the importance of framing AI-supported tools in ways that preserve learners' sense of autonomy and agency (Reeve, 2012).

In physical education, where student motivation is closely linked to participation and persistence, these dynamics are particularly salient. Empirical studies show that motivational climates emphasizing mastery, improvement, and supportive feedback are associated with higher levels of engagement in PE classes (Fredricks et al., 2004). Accordingly, AI-supported feedback systems may enhance engagement when they are integrated within pedagogical approaches that prioritize individual progress rather than normative comparison.

### 3.5 Research in developing and underrepresented contexts

A recurrent limitation in the literature on artificial intelligence in education concerns the overrepresentation of studies conducted in technologically advanced and resource-rich contexts. Empirical investigations in developing countries remain comparatively scarce, despite growing global interest in educational technologies (Trucano and Trucano, 2016). This imbalance raises concerns regarding the generalizability of findings and the transferability of pedagogical models developed in high-income settings.

Available research suggests that structural constraints such as limited infrastructure, uneven access to resources, and institutional culture significantly shape the outcomes of technology integration in education. In the context of physical education, similar constraints have been documented across public secondary schools in resource-constrained educational systems, influencing both instructional practices and student engagement (Kirk, 2009; Trucano and Trucano, 2016). Complementary analyses further show that pedagogical approaches and classroom climate play a critical role in shaping learners' experiences in physical education, underscoring the importance of context-sensitive and autonomy-supportive instructional practices (Fredricks et al., 2004; Reeve, 2012).

International policy frameworks increasingly call for more inclusive and contextually grounded research agendas in the field of AI in education. By documenting empirical findings from underrepresented contexts, researchers can contribute to a more nuanced understanding of how AI-supported pedagogical innovations interact with local educational ecologies (Miao and Holmes, 2021; Bo, 2025).

### 3.6 Summary and research positioning

The empirical literature reviewed highlights three central insights relevant to the present study. First, AI-supported learning environments can enhance learning outcomes when they are grounded in pedagogical theory and mediated by teacher expertise, rather than implemented as stand-alone technological solutions (Zawacki-Richter et al., 2019). Second, empirical research on AI integration in physical education—particularly at the secondary school level—remains limited, creating a need for contextually grounded investigations that extend beyond higher education and elite sport contexts (Koekoek and Van Hilvoorde, 2019). Third, motivational and engagement-related processes play a crucial mediating role in shaping the educational impact of AI-supported instruction, reinforcing the relevance of educational psychology frameworks in interpreting learning outcomes (Ryan and Deci, 2000).

Positioned at the intersection of AI in education, motor learning theory, and motivational research, the present study addresses these gaps by examining AI-supported instruction in secondary school physical education within a developing country context. By mobilizing validated instruments and a quasi-experimental design, the study contributes empirically grounded evidence to international debates on human-centered AI integration in education (Crompton and Burke, 2023).

## 4 Methodology

### 4.1 Research design

This study adopted a quantitative, quasi-experimental pre-test/post-test design involving an experimental group and a control group (Cohen et al., 2018). Quasi-experimental designs are particularly appropriate in school-based research contexts where random assignment of participants is often impractical due to institutional and organizational constraints (Shadish, 2002). By comparing outcomes across intact classes exposed to different instructional conditions, this design allows for the examination of intervention effects while preserving ecological validity in authentic educational settings (Creswell and Creswell, 2014).

To mitigate threats to internal validity, baseline equivalence between groups was established through pre-test comparisons across all outcome variables. In addition, both groups followed identical curricular objectives, lesson durations, and instructional schedules throughout the intervention period, ensuring that observed differences could be attributed primarily to the AI-supported instructional approach rather than to curricular variation or exposure time (Holmes et al., 2022).

The use of intact classes reflected organizational constraints within the participating school and was intended to preserve the natural instructional context. Consequently, individual random assignment was not feasible. This design favors ecological validity but limits the ability to draw causal inferences.

### 4.2 Context and participants

The study was conducted in a public secondary school located in an urban area of Tunisia during the second academic term. The participating school was selected through convenience sampling based on administrative authorization and willingness to participate in the study. Two existing classes were included because reorganizing students into randomly assigned groups was not feasible within the school's organizational structure. Physical education lessons were delivered in accordance with the national curriculum, with two sessions per week for each class. This instructional context reflects typical conditions in Tunisian public secondary schools, characterized by relatively large class sizes and limited material resources (Darwez et al., 2020; Saaidia et al., 2026).

A total of 56 students participated in the study (age range: 15–17 years; *M* = 16.4, SD = 0.9). Participants were distributed across two intact classes assigned to either the experimental group (AI-supported instruction; *n* = 28) or the control group (traditional instruction; *n* = 28). As shown in Table 1, the two groups were comparable at baseline in terms of age, gender distribution, prior physical education achievement, and attendance rate. Independent-samples *t*-tests conducted on pre-test measures further confirmed the absence of statistically significant differences between groups across motor learning, intrinsic motivation, and engagement variables (*p* > 0.05), indicating baseline equivalence in line with methodological recommendations for quasi-experimental research (Field, 2018).

**Table 1:** Demographic and baseline characteristics of participants.

Variable	Experimental group (*n* = 28)	Control group (*n* = 28)
Mean age (years)	16.3 ± 0.9	16.5 ± 0.8
Gender (boys/girls)	14/14	15/13
Prior PE achievement (average)	11.5 ± 1.3	11.6 ± 1.2
Attendance rate (%)	94.2	93.7

Independent-samples *t*-tests revealed no statistically significant differences between groups at pre-test (*p* > 0.05), indicating baseline equivalence.

**Figure 1:**
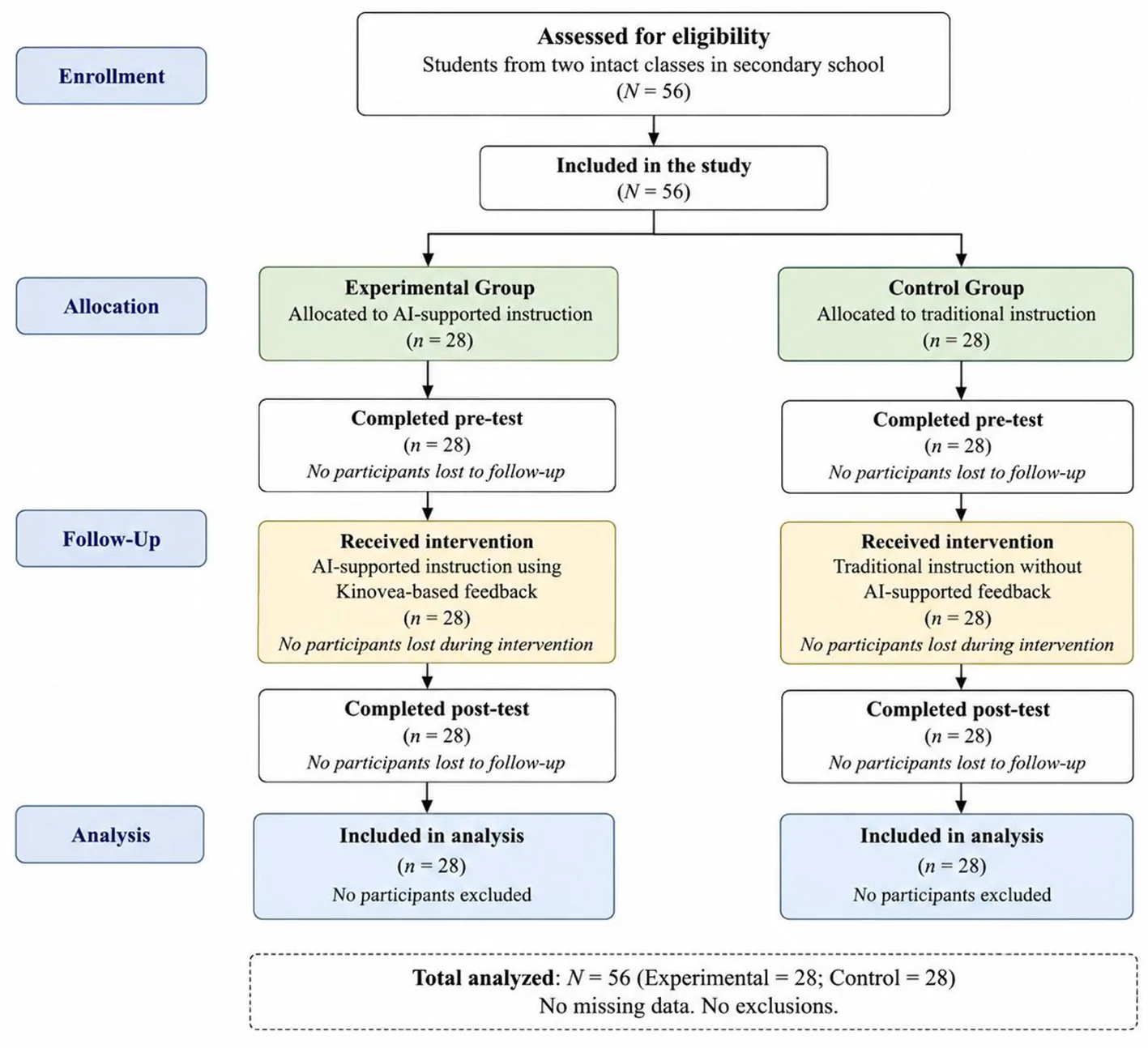
Flow diagram of participant recruitment, group allocation, intervention, and analysis.

Eligibility criteria included regular enrollment in the participating school, attendance in scheduled physical education lessons, and the absence of medical restrictions preventing participation in physical activity. Students who were absent during either the pre-test or post-test assessments or who did not complete all study procedures were excluded from the final analyses. No participant withdrew from the study during the intervention period. Consequently, all students who met the eligibility criteria completed both the pre-test and post-test assessments and were included in the final analyses. All 56 enrolled students completed both the pre-test and post-test assessments. No participant withdrew from the study, and no missing data were identified. Consequently, all participants were included in the final statistical analyses. The flow of participant recruitment, group allocation, intervention, and final analysis is summarized in Figure 1.

### 4.3 Instructional intervention

The instructional intervention was implemented over an 8-week period, comprising 16 physical education sessions for each group. Instructional content focused on fundamental motor skills drawn from athletics and gymnastics units, which constitute core components of the secondary school PE curriculum (Kirk, 2009). To ensure comparability across conditions, lesson objectives, duration, and assessment criteria were standardized for both groups, with only the mode of feedback and instructional support differing between the experimental and control conditions.

#### 4.3.1 Experimental group: AI-supported instruction

Students in the experimental group received AI-supported instruction using Kinovea (Version 0.9.5), an open-source software for computer-assisted motion analysis. Students' performances were recorded using a smartphone equipped with a Full HD (1,920 × 1,080 pixels) camera operating at 30 frames per second. The smartphone was mounted on a tripod positioned approximately 4–5 m from the performance area at a height of 1.20 m, providing a standardized lateral view of each performance. The camera position, recording distance, and lighting conditions were kept constant throughout the intervention to ensure standardized data collection across all participants. Each student completed three attempts for every motor task, and the best performance was selected for immediate analysis. Kinovea enabled frame-by-frame movement analysis, allowing comparison between students' performance and the expected movement pattern. Immediately after each attempt (within approximately 30–60 s), students reviewed their recorded performance with the teacher using Kinovea. The software highlighted predefined movement indicators, after which the teacher interpreted the results and provided individualized corrective feedback before the next practice trial. The teacher interpreted the software outputs and delivered individualized corrective feedback before the subsequent practice trial. The software served exclusively as a formative instructional support tool and did not automatically evaluate student performance or generate grades. All pedagogical decisions, instructional explanations, and corrective feedback remained under the responsibility of the physical education teacher, in accordance with human-centered AI principles (Floridi et al., 2018). The AI-supported application was used exclusively as a formative instructional aid. It did not automatically evaluate student performance or generate grades. Rather, it highlighted predefined movement characteristics that were subsequently interpreted by the teacher during instructional feedback, consistent with human-centered AI principles emphasizing pedagogical mediation and transparency (Floridi et al., 2018).

**Figure 2:**
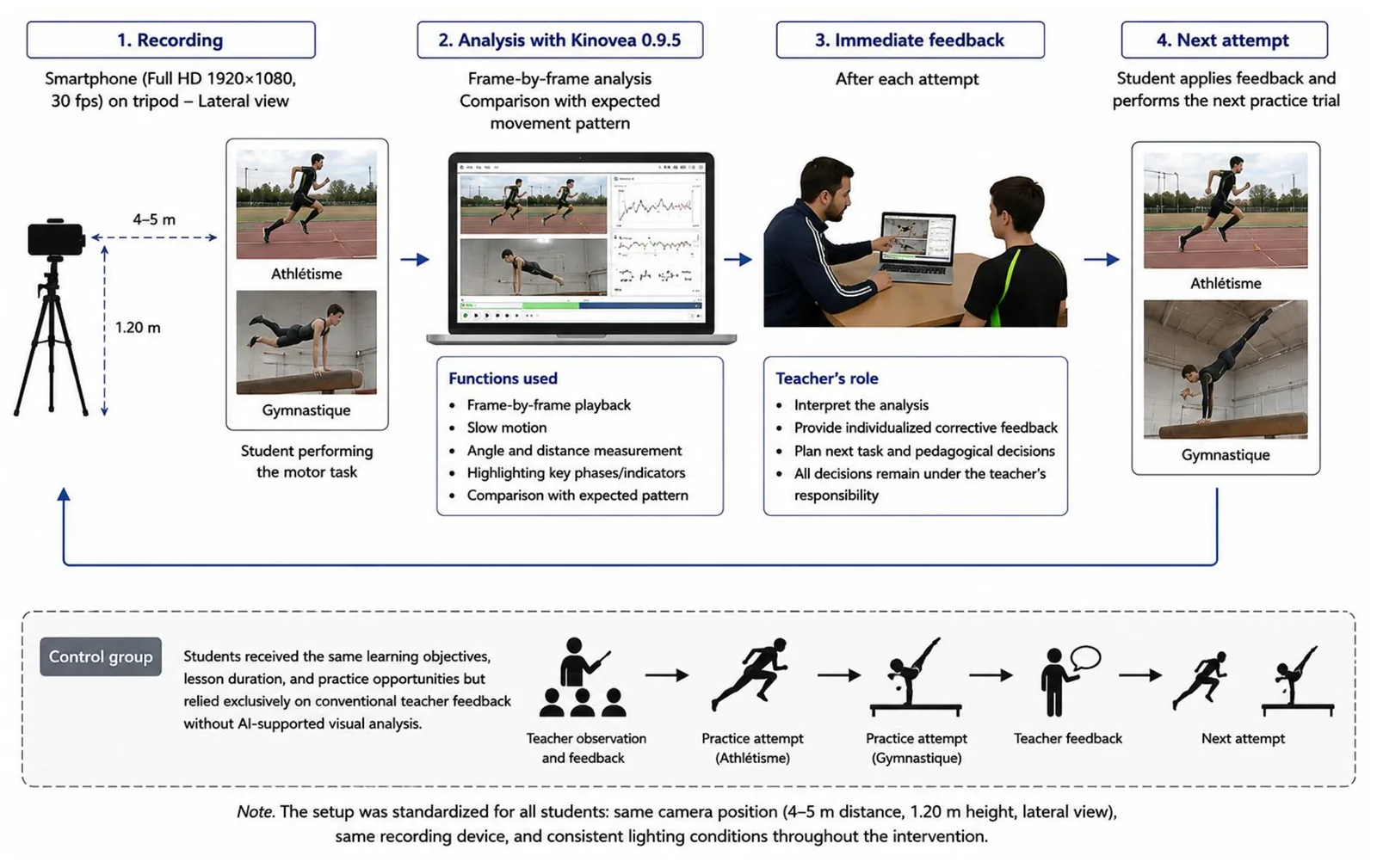
Experimental setup and AI-supported instructional workflow in physical education.

Feedback focused on core aspects of movement execution, such as body alignment, coordination, and timing, and was used to support learners' reflective practice and self-regulation processes (Schmidt et al., 2018). By making performance criteria more visible, AI-supported feedback complemented teachers' observational feedback and facilitated more differentiated instructional support across heterogeneous learner profiles. The teacher remained responsible for lesson planning, task demonstrations, instructional explanations, and pedagogical decisions. AI-generated information served only as an additional source of formative feedback and never replaced the teacher's professional judgment. The experimental setup and AI-supported instructional workflow are illustrated in Figure 2.

In contrast, students assigned to the control group followed the same curricular objectives, learning tasks, session duration, and practice schedule as those in the experimental group throughout the 8-week intervention. Instruction was delivered by the same physical education teacher to ensure consistency in teaching experience and classroom management. Students practiced the same motor tasks and completed the same number of practice trials under identical environmental conditions. Following each practice attempt, the teacher provided conventional pedagogical feedback based on direct observation, including verbal explanations, demonstrations, and corrective instructions when necessary. No AI-supported video analysis, movement visualization, or digital performance indicators were used. Consequently, any differences between groups can reasonably be attributed to the integration of AI-supported visual feedback rather than to differences in instructional content, practice time, or teacher involvement.

### 4.4 Instruments and measures

To ensure methodological rigor, validated instruments were employed to assess motor learning, intrinsic motivation, and student engagement. The selection of measurement tools was guided by their established psychometric properties and relevance to physical education research.

Motor learning was assessed using the Test of Gross Motor Development–Third Edition (TGMD-3), a widely validated instrument for assessing gross motor skills in children and adolescents (Ulrich, 2019). Previous studies have reported excellent psychometric properties, with internal consistency coefficients ranging from 0.85 to 0.94 (Mohammadi et al., 2019). Previous studies have also demonstrated strong construct validity for the TGMD-3 across school-aged populations. In the present study, the TGMD-3 demonstrated excellent internal consistency (Cronbach's α = 0.881) across the 13 performance criteria. Inter-rater reliability was also excellent [ICC = 0.951, 95% CI (0.918, 0.973), two-way random-effects model, absolute agreement, *F* = 20.87, *p* < 0.001], confirming the consistency of motor performance ratings. TGMD-3 scores were calculated according to the standardized scoring procedures described in the test manual, with higher scores indicating better motor performance.

Intrinsic motivation was assessed using the Intrinsic Motivation Inventory (IMI), a validated multidimensional instrument grounded in Self-Determination Theory (Ryan and Deci, 2000). Previous research has reported Cronbach's alpha coefficients generally ranging between 0.80 and 0.90 across the IMI subscales. The IMI has also demonstrated satisfactory construct and convergent validity in educational research. In the present sample, the IMI demonstrated good internal consistency (Cronbach's α = 0.85).

The questionnaire was administered in its adapted version used for the study. Item scores were averaged to obtain an overall intrinsic motivation score, with higher values indicating greater intrinsic motivation.

Student engagement was assessed using a multidimensional Student Engagement Scale measuring behavioral, cognitive, and affective engagement (Fredricks et al., 2004). Previous validation studies have reported good internal consistency as well as satisfactory construct validity across the behavioral, cognitive, and affective dimensions (Henrie et al., 2015). In the present study, the overall engagement scale demonstrated good internal consistency (Cronbach's α = 0.87).

Engagement scores were computed by averaging responses across the behavioral, cognitive, and affective dimensions. The previous wording referring to percentage-based indices has been corrected to accurately reflect the scoring procedure, with higher scores indicating greater student engagement.

### 4.5 Data collection procedure

Data collection was conducted in three successive phases to ensure methodological consistency across measurement points. First, during the pre-test phase, all instruments were administered 1 week prior to the intervention under standardized testing conditions. This baseline assessment established initial equivalence between groups and provided reference points for subsequent comparisons (Creswell and Creswell, 2014).

Both the experimental and control groups completed the pre-test and post-test assessments under identical conditions, using the same assessment protocols, facilities, equipment, and testing schedule.

Second, during the intervention phase, AI-supported or traditional instruction was implemented over the 8-week period in accordance with the predefined instructional protocol. Throughout this phase, fidelity of implementation was monitored by the teacher-researcher to ensure that instructional objectives and lesson structures remained comparable across groups, consistent with recommended practices for classroom-based intervention research (Aleven et al., 2016).

To ensure procedural consistency, all instructional sessions followed the same timetable, lesson duration, and curricular objectives. The only difference between groups concerned the integration of AI-supported feedback in the experimental condition.

Finally, the post-test phase involved re-administration of all instruments under conditions identical to those of the pre-test phase. This procedural consistency supported the internal validity of pre- to post-intervention comparisons (Field, 2018). To minimize measurement bias, all assessments were conducted by the same trained assessor following standardized administration procedures.

Standardized assessment procedures were applied consistently across all participants to reduce measurement variability. The use of the same assessor throughout the study further contributed to procedural consistency.

Because the intervention was conducted in an authentic school setting, assessor blinding was not implemented. This may have introduced a risk of detection bias and should therefore be considered when interpreting the findings.

### 4.6 Statistical analysis

Statistical analyses were performed using IBM SPSS Statistics (Version 26) (International Business Machines Corporation, Armonk, NY, USA) (Andy, 2018). Descriptive statistics (means and standard deviations) were calculated for all variables. Baseline equivalence between groups was assessed using independent-samples *t*-tests. Within-group changes from pre-test to post-test were analyzed using paired-samples *t*-tests, whereas between-group comparisons at pre-test and post-test were conducted using independent-samples *t*-tests. Prior to analysis, the assumptions of normality and homogeneity of variance were examined using the Shapiro–Wilk test and Levene's test, respectively. Cohen's *d* was calculated to estimate effect sizes and interpreted according to conventional criteria (Cohen, 1988; Lakens, 2013). Statistical significance was set at *p* < 0.05. Although mixed-design ANOVA or linear mixed-effects models are generally recommended for repeated-measures quasi-experimental designs, the present study retained the planned analytical strategy based on *t*-tests because of the modest sample size and the exploratory nature of this school-based investigation. Accordingly, the findings should be interpreted with appropriate caution. Future studies with larger samples should employ mixed-model approaches together with appropriate corrections for multiple comparisons.

### 4.7 Ethical considerations

The study was conducted in accordance with established ethical standards for educational research. Institutional authorization was obtained prior to data collection, and informed consent was secured from all participating students and their legal guardians. Participation was voluntary, and students were informed of their right to withdraw from the study at any stage without academic consequences.

All data were anonymized and used exclusively for research purposes. Particular attention was given to issues of data privacy, transparency, and responsible use of AI-generated information, consistent with ethical guidelines for learning analytics and AI in education (Pardo and Siemens, 2014). The design and implementation of the AI-supported tools adhered to human-centered AI principles emphasizing transparency, accountability, and respect for learners' rights (Floridi et al., 2018; Holmes et al., 2022). Because the physical education teacher also participated in implementing the intervention and collecting the assessment data, particular attention was paid to minimizing any perception of coercion or undue influence. Participation was entirely voluntary, students and their legal guardians were informed that participation or non-participation would have no consequences for academic evaluation, and all collected data were anonymized before analysis. This dual role is nevertheless acknowledged as a methodological limitation and should be considered when interpreting the findings.

## 5 Results

### 5.1 Preliminary analyses

Prior to inferential analyses, preliminary data screening procedures were conducted to assess data quality and the suitability of parametric tests. Examination of skewness and kurtosis values indicated that distributions for motor learning, intrinsic motivation, and student engagement were within acceptable ranges, supporting the use of *t*-tests for subsequent analyses (Field, 2018). In addition, no extreme outliers were detected that would warrant exclusion or transformation procedures.

Baseline equivalence between the experimental and control groups was examined using independent-samples *t*-tests on pre-test scores across all outcome variables. Results confirmed the absence of statistically significant differences between groups at baseline (*p* > 0.05), satisfying key methodological requirements for quasi-experimental designs in educational research (Shadish, 2002). Tables 2 and 3 present the demographic characteristics and baseline equivalence between the experimental and control groups.

**Table 2:** Demographic characteristics of the sample (*N* = 56).

Group	*N*	Mean age (SD)	Min age	Max age	Boys *n* (%)	Girls *n* (%)
Experimental	28	16.4 (0.9)	15	17	14 (50.0%)	14 (50.0%)
Control	28	16.5 (0.8)	15	17	13 (46.4%)	15 (53.6%)
Total	56	16.45 (0.85)	15	17	27 (48.2%)	29 (51.8%)

SD, standard deviation.

**Table 3:** Baseline equivalence at pre-test between groups.

Outcome	Group	Pre-test mean	SD	*t* (df = 54)	*p*
Motor learning	Experimental	11.50	1.30		
	Control	11.60	1.20	0.31	0.76
Intrinsic motivation	Experimental	3.02	0.41		
	Control	3.08	0.38	0.56	0.58
Student engagement %	Experimental	62.10	6.40		
	Control	61.70	6.10	0.24	0.81

Independent-samples *t*-tests; *p* > 0.05 indicates baseline equivalence.

### 5.2 Effects of AI-supported instruction on motor learning

Motor learning outcomes were assessed using TGMD-3 total scores. Descriptive statistics indicated comparable pre-test scores between the experimental and control groups, confirming baseline equivalence in motor skill levels. Following the 8-week intervention, the experimental group demonstrated substantially higher post-test scores relative to the control group, reflecting more pronounced improvements in movement quality.

Paired-samples *t*-tests revealed a statistically significant improvement in motor learning for students receiving AI-supported instruction, with a very large effect size [*t*_(27)_ = 14.86, *p* < 0.001, *d* = 1.95]. Although the control group also exhibited a statistically significant pre- to post-test improvement, the magnitude of change was comparatively modest [*t*_(27)_ = 3.12, *p* < 0.01, *d* = 0.42]. These findings indicate that the integration of AI-supported feedback contributed to accelerated motor skill acquisition beyond gains associated with routine practice alone. The very large effect size (*d* = 1.95) indicates that these improvements were not only statistically significant but also educationally meaningful, highlighting the practical value of AI-supported feedback for motor skill acquisition. Tables 4 and 5 present the between-group post-test results and within-group pre-post comparisons for motor learning.

**Table 4:** Post-test motor learning outcomes and between-group comparison.

Group	Post-test mean	SD	*t* (df = 54)	*p*	Cohen's *d*
Experimental	16.20	1.40			
Control	12.70	1.10	6.48	<0.001	1.95

Independent-samples *t*-test; *d* = Cohen's effect size.

**Table 5:** Within-group pre–post comparisons for motor learning.

Group	Pre-test mean (SD)	Post-test mean (SD)	*t* (df = 27)	*p*	Cohen's *d*
Experimental	11.50 (1.30)	16.20 (1.40)	14.86	<0.001	1.95
Control	11.60 (1.20)	12.70 (1.10)	3.12	<0.01	0.42

Paired-samples *t*-tests within each group.

### 5.3 Effects of AI-supported instruction on intrinsic motivation

Intrinsic motivation scores, measured using the Intrinsic Motivation Inventory, followed divergent trajectories across the two groups. While both groups reported comparable levels of intrinsic motivation at pre-test, post-test scores indicated a marked increase among students exposed to AI-supported instruction.

Paired-samples *t*-tests demonstrated a statistically significant increase in intrinsic motivation within the experimental group [*t*_(27)_ = 8.94, *p* < 0.001, *d* = 1.10]. In contrast, changes observed in the control group did not reach conventional levels of statistical significance (*p* = 0.06) and were associated with a small effect size (*d* = 0.28), suggesting limited practical relevance. The large effect size observed in the experimental group (*d* = 1.10) suggests that AI-supported instruction had a substantial positive influence on students' intrinsic motivation toward physical education. Tables 6 and 7 present the between-group post-test results and within-group pre-post comparisons for intrinsic motivation.

**Table 6:** Post-test intrinsic motivation outcomes and between-group.

Group	Post-test mean	SD	*t* (df = 54)	*p*	Cohen's *d*
Experimental	4.12	0.48			
Control	3.29	0.40	7.21	<0.001	1.10

Independent-samples *t*-test.

**Table 7:** Within-group pre–post comparisons for intrinsic motivation.

Group	Pre-test mean (SD)	Post-test mean (SD)	*t* (df = 27)	*p*	Cohen's *d*
Experimental	3.02 (0.41)	4.12 (0.48)	8.94	<0.001	1.10
Control	3.08 (0.38)	3.29 (0.40)	1.96	0.06	0.28

Paired-samples *t*-tests within each group.

### 5.4 Effects on student engagement

Student engagement levels were examined using percentage-based indices derived from the Student Engagement Scale, capturing behavioral, cognitive, and affective dimensions of engagement. Descriptive statistics indicated comparable baseline engagement levels across groups, while post-test scores revealed a substantial divergence favoring the experimental condition.

Independent-samples *t*-tests conducted on post-test engagement scores showed a statistically significant difference between groups [*t*_(54)_ = 6.87, *p* < 0.001], with a large effect size (*d* = 1.42). Students who experienced AI-supported instruction demonstrated markedly higher levels of engagement than their peers in the control group. The large effect size (*d* = 1.42) further demonstrates that the intervention produced educationally meaningful improvements in student engagement throughout the instructional period. Tables 8 and 9 present the between-group post-test results and within-group pre-post comparisons for student engagement.

**Table 8:** Post-test student engagement outcomes and between-group comparison.

Group	Post-test (mean ±SD)
Experimental (*n* = 28)	86.10 ± 5.80
Control (*n* = 28)	69.20 ± 6.30

Independent-samples *t*-test: *t*_(54)_ = 6.87, *p* < 0.001, Cohen's *d* = 1.42.

Post-test student engagement scores were compared between the experimental and control groups using an independent-samples *t*-test. Values are presented as mean ± standard deviation (SD). The experimental group demonstrated significantly higher engagement than the control group following the intervention.

**Table 9:** Within-group pre–post comparisons for student engagement.

Group	Pre-test (mean ±SD)	Post-test (mean ±SD)	Mean difference	*t* (df = 27)	*p*	Cohen's *d*
Experimental (*n* = 28)	62.10 ± 6.40	86.10 ± 5.80	24.00	11.72	<0.001	1.42
Control (*n* = 28)	61.70 ± 6.10	69.20 ± 6.30	7.50	2.10	0.045	0.60

Student engagement scores were compared between pre-test and post-test within each group using paired-samples *t*-tests. Values are presented as mean ± standard deviation (SD). Mean differences, *t* statistics, *p* values, and Cohen's *d* effect sizes are reported. The experimental group showed a statistically significant and substantial improvement in student engagement following AI-supported instruction, whereas the control group showed only a modest improvement.

### 5.6 Summary of results

Overall, the results demonstrate that AI-supported instruction in physical education led to substantially greater improvements in motor learning, intrinsic motivation, and student engagement compared with traditional instruction. Because all enrolled participants completed both measurement occasions, the analyses included the entire study sample without participant attrition or missing post-test data. The magnitude of observed effect sizes indicates not only statistical significance but also strong educational relevance, supporting interpretations of practical significance in educational research (Cohen, 2013).

Taken together, these findings suggest that AI-supported feedback, when embedded within teacher-mediated instructional designs, can enhance learning processes without undermining the embodied and relational dimensions of physical education. The convergence of improvements across performance, motivational, and engagement-related outcomes provides a robust empirical foundation for the subsequent discussion of pedagogical implications and theoretical contributions.

## 6 Discussion

### 6.1 Overview of the main findings

The present study examined whether integrating AI-supported instructional tools into secondary school physical education could enhance students' motor learning, intrinsic motivation, and engagement. Because the study employed a quasi-experimental design using intact classes, the findings should be interpreted as evidence of association rather than definitive causal effects. The results provide converging evidence that AI-supported instruction, when pedagogically mediated by the teacher, is associated with statistically significant and educationally meaningful improvements across all three outcome variables.

These findings resonate with contemporary research emphasizing that the educational impact of artificial intelligence depends less on technological sophistication than on its integration within coherent pedagogical designs. In line with learning-oriented perspectives on AI in education, the present results suggest that AI-supported tools function most effectively as complements to teacher-led instruction, reinforcing rather than displacing pedagogical practices (Luckin and Holmes, 2016).

From an educational psychology standpoint, the convergence of performance-, motivation-, and engagement-related gains underscores the interdependence of cognitive and affective processes in learning. By enhancing feedback quality and making performance criteria more visible, AI-supported instruction may have contributed to learners' sense of competence and agency, thereby supporting more sustained engagement in practice activities. This interpretation is consistent with broader theoretical frameworks linking feedback processes to motivational regulation (Hattie and Timperley, 2007). Nevertheless, because the study employed a quasi-experimental design based on intact classes, the findings should be interpreted with appropriate caution. Although the observed effect sizes were large across the three outcome variables, they should not be attributed exclusively to the AI-supported intervention itself. Several contextual and methodological factors may also have contributed to the magnitude of these effects, including novelty effects associated with the introduction of AI-supported instruction, increased student attention resulting from participation in an educational innovation, demand characteristics, teacher expectancy effects, social desirability in self-reported measures, regression to the mean, and the absence of individual random assignment. Consequently, the observed effect sizes should be interpreted as reflecting the combined influence of the AI-supported instructional approach and the authentic educational context in which it was implemented, rather than as definitive evidence of the technology alone. Future randomized and multi-site studies are needed to determine whether similar effect sizes can be replicated under more rigorous experimental conditions.

### 6.2 AI-supported feedback and motor learning

The most pronounced effects observed in this study concern motor learning outcomes, with students in the experimental group demonstrating substantially greater improvements in TGMD-3 scores than their peers receiving traditional instruction. From a motor learning perspective, these findings are consistent with theoretical models emphasizing the role of augmented feedback in facilitating movement refinement and skill acquisition (Schmidt et al., 2018). It is also noteworthy that students in the control group demonstrated modest improvements in motor learning over the intervention period. Such gains are expected in school-based physical education and may reflect the combined influence of regular practice, natural skill development, repeated exposure to the assessed tasks, and teacher guidance. These findings suggest that learning occurred in both instructional conditions, while the larger improvements observed in the AI-supported group indicate that the technology-enhanced, teacher-mediated feedback may have provided additional educational benefits beyond those associated with conventional instruction alone.

Within the challenge point framework, optimal learning occurs when task difficulty and informational feedback are appropriately matched to learners' current skill levels. The AI-supported feedback employed in this study may have contributed to optimizing this balance by providing timely, individualized cues that supported error detection and movement correction (Guadagnoli and Lee, 2004). Importantly, these benefits appear to stem not from automation *per se*, but from the pedagogical mediation of AI-generated information by the teacher. These findings are also consistent with previous research indicating that technology-enhanced feedback is most effective when integrated into pedagogically meaningful learning environments rather than used as a standalone technological solution (Zawacki-Richter et al., 2019; Holmes et al., 2022).

Educational psychology research further suggests that feedback effectiveness depends on learners' capacity to interpret and integrate performance-related information into their practice. In this respect, the teacher's role in contextualizing AI-supported feedback likely played a critical role in translating technological outputs into meaningful learning cues, echoing findings from learning analytics research highlighting the importance of guided sense-making processes (Wise and Shaffer, 2015).

### 6.3 Motivation, perceived competence, and engagement

Beyond performance gains, the significant increase in intrinsic motivation observed among students exposed to AI-supported instruction provides insight into the motivational mechanisms underpinning learning outcomes. According to self-determination theory, perceived competence constitutes a central driver of intrinsic motivation, particularly in performance-oriented learning contexts such as physical education (Ryan and Deci, 2000). By making progress visible and clarifying performance criteria, AI-supported feedback may have strengthened students' perceptions of competence, thereby supporting more positive motivational trajectories.

At the same time, motivational benefits are not inherent to the use of technology. Critical perspectives caution that digital tools may undermine autonomy when they are perceived as controlling or evaluative mechanisms rather than as resources for learning (Selwyn, 2019). The present findings suggest that the human-centered framing of AI-supported feedback as a formative aid rather than a surveillance device may have contributed to preserving students' sense of autonomy and fostering engagement.

Engagement gains observed in the experimental group further reflect the interdependence of motivational and behavioral processes in learning. Educational psychology research conceptualizes engagement as a multidimensional construct encompassing behavioral, cognitive, and affective components, all of which are sensitive to classroom climate and instructional design (Fredricks et al., 2004). By supporting perceived competence and providing timely feedback, AI-supported instruction may have contributed to a more participatory and focused learning environment, consistent with findings on engagement in technology-enhanced contexts (Reeve, 2012). Taken together, these findings suggest that AI-supported instruction may promote learning not only by improving performance feedback but also by fostering motivational processes that sustain students' active participation in physical education.

### 6.4 Teacher mediation and human-centered AI

A central contribution of this study lies in highlighting the mediating role of the teacher in AI-supported physical education. Rather than functioning as autonomous instructional agents, the AI-supported tools used in this study operated as mediating artifacts whose pedagogical value depended on how teachers selected, interpreted, and framed performance-related information. This finding aligns with human-centered AI frameworks emphasizing that educational technologies should augment teachers' professional judgment rather than replace it (Floridi et al., 2018).

Research in learning analytics similarly underscores that data become educationally meaningful only when educators actively engage in interpretive processes and integrate insights into instructional decision-making (Pardo and Siemens, 2014). In physical education, where learning is embodied and context-dependent, the teacher's interpretive role is particularly salient. The present findings suggest that AI-supported feedback can enhance teachers' observational capacity, but its pedagogical effectiveness ultimately rests on professional expertise and pedagogical alignment (Holmes et al., 2022).

### 6.5 Interpreting the findings in the Tunisian context

Interpreting the present findings within the Tunisian secondary school context provides important insights into the situated nature of AI integration in physical education. Public schools in Tunisia operate under structural constraints, including large class sizes, limited access to technological resources, and strong curricular demands, which shape both teaching practices and learning opportunities (Kirk, 2009). Within such conditions, providing individualized feedback to all students represents a persistent pedagogical challenge.

The observed learning gains associated with AI-supported instruction suggest that even relatively simple technological tools, when pedagogically aligned, can support more differentiated feedback practices. This finding resonates with analyses emphasizing that educational innovation in resource-constrained contexts depends less on technological sophistication than on contextual appropriateness and pedagogical coherence (Trucano and Trucano, 2016).

Moreover, the motivational and engagement-related benefits observed in this study may be particularly relevant in contexts where student disengagement in physical education has been documented. Prior research conducted in secondary school physical education contexts highlights substantial variations in student participation patterns and the decisive role of classroom climate in shaping engagement (Fredricks et al., 2004; Ennis, 2017). The present findings suggest that AI-supported feedback, when framed as a formative learning aid, may contribute to more inclusive and engaging PE environments even under structural constraints.

### 6.6 Positioning the study within international research

When situated within the broader international literature on AI in education, the present findings both corroborate and extend existing evidence. Consistent with prior meta-analytic reviews, the results indicate that AI-supported instruction can enhance learning outcomes when embedded within sound pedagogical designs and mediated by teacher expertise (Zawacki-Richter et al., 2019).

It should also be acknowledged that the present quasi-experimental study provides weaker support for causal inference than randomized controlled trials and meta-analytic syntheses. Consequently, comparisons with findings from studies employing stronger experimental designs should be interpreted cautiously, and the present results should be regarded as complementary contextual findings rather than equivalent causal evidence.

At the same time, the study extends this literature by focusing on a practice-based discipline and a secondary school context that remain underrepresented in AI in education research. By examining AI-supported instruction in secondary school physical education, the present study responds to calls for greater disciplinary diversity in AI in education research (Crompton and Burke, 2023).

Furthermore, by providing empirical evidence from a developing country context, the study contributes to a more globally inclusive evidence base, in line with international policy frameworks emphasizing the need for context-sensitive research (Miao and Holmes, 2021). Accordingly, the present study contributes to the growing body of evidence supporting human-centered approaches to AI integration, emphasizing that educational benefits depend on pedagogical design, teacher mediation, and contextual adaptation rather than on technology alone.

### 6.7 Theoretical implications

From a theoretical perspective, the present study empirically links AI-supported feedback with motor learning theory, self-determination theory, and distributed cognition frameworks. The observed improvements in motor learning align with models emphasizing the role of feedback and practice conditions in skill acquisition (Schmidt and Lee, 2019), while motivational gains can be interpreted through the lens of self-determination theory (Ryan and Deci, 2000).

By situating AI within a distributed cognition framework, the study underscores that learning emerges from interactions among learners, teachers, and technological artifacts rather than from technological inputs alone (Salomon, 1997), reinforcing human-centered perspectives on AI in education (Holmes et al., 2022).

### 6.8 Summary of the discussion

In summary, AI-supported feedback can significantly enhance motor learning in physical education when integrated within teacher-mediated instructional designs (Guadagnoli and Lee, 2004). Motivational and engagement-related benefits are closely tied to how AI tools are framed and experienced by learners (Deci and Ryan, 2008), while contextual factors shape the outcomes of AI integration in practice (Trucano and Trucano, 2016).

## 7 Limitations, implications, and future research

### 7.1 Methodological limitations

Despite the robustness of the quasi-experimental design and the use of validated instruments, several methodological limitations warrant consideration when interpreting the findings. First, the absence of random assignment limits the strength of causal inferences that can be drawn from the results. Although baseline equivalence between groups was established and standard controls were implemented, unmeasured contextual variables such as classroom climate, peer dynamics, or prior exposure to digital tools may have influenced learning outcomes (Shadish, 2002).

An additional methodological limitation concerns the assessment procedure. Although all assessments were conducted by the same trained assessor using standardized administration procedures to ensure consistency, assessor blinding was not implemented. Consequently, the possibility of observer bias cannot be completely excluded and should be considered when interpreting the findings. Second, the sample size, while consistent with school-based intervention research, constrains the generalizability of the findings. Larger, multi-site samples would enable more nuanced analyses of subgroup effects and enhance external validity (Field, 2018). Third, the intervention duration was limited to 8 weeks, which restricts conclusions regarding the long-term retention and transfer of motor skills. Motor learning research emphasizes the importance of delayed retention tests for assessing the stability of learning effects over time (Schmidt et al., 2018).

Finally, the reliance on quantitative measures, although appropriate for examining outcome variables, provides limited insight into the qualitative dimensions of learners' and teachers' experiences with AI-supported instruction. Although baseline equivalence was established across the measured variables, the absence of individual random assignment means that residual selection bias and unmeasured confounding variables cannot be excluded. Factors such as prior motivation, familiarity with digital technologies, or broader academic engagement may have differed between groups and could have influenced the observed associations. Consequently, the findings should be interpreted as evidence of association rather than definitive causal effects. Future studies employing randomized designs or advanced analytical approaches should further evaluate the potential influence of unmeasured confounding.

Another limitation concerns the absence of objective individual-level AI usage data. Although all students assigned to the experimental condition participated in the planned instructional activities, no platform usage logs or comparable analytics were collected to quantify individual exposure to the AI-supported tool. Consequently, the present study evaluates the educational outcomes associated with assignment to the AI-supported instructional condition rather than the specific effects of individual AI use. Future studies should incorporate objective usage data to examine possible dose–response relationships and intervention fidelity.

An additional limitation is that no formal robustness or sensitivity analyses (e.g., non-parametric analyses, influence diagnostics, or alternative analytical models) were conducted. Although preliminary assumption checks supported the use of parametric tests, future studies should evaluate the stability of findings using complementary analytical approaches to strengthen confidence in the robustness of the reported results.

### 7.2 Pedagogical implications for physical education

The findings of this study yield several pedagogical implications for secondary school physical education. First, AI-supported tools appear most effective when employed to enhance formative feedback processes rather than to automate evaluation or grading. This observation aligns with research emphasizing that feedback should support learning and self-regulation rather than function primarily as a control mechanism (Hattie and Timperley, 2007).

Second, the results underscore the central role of teacher mediation in the effective use of AI-supported feedback. Teachers' professional judgment is essential for selecting relevant performance indicators, interpreting AI-generated information, and aligning feedback with learners' developmental needs (Wise and Shaffer, 2015). This suggests that teacher education and professional development programs should integrate training on pedagogically grounded uses of AI in physical education contexts.

Third, AI-supported instruction may contribute to addressing motivational challenges commonly observed in adolescent PE by supporting learners' perceived competence and making progress visible. When framed as a learning aid rather than an evaluative instrument, AI-supported feedback can foster engagement without compromising learners' sense of autonomy, consistent with motivational principles articulated in self-determination theory (Ryan and Deci, 2000).

### 7.3 Institutional and policy implications

At the institutional and policy levels, the findings suggest that meaningful AI integration in physical education does not necessarily require highly sophisticated or costly technologies. Relatively simple AI-supported tools, when pedagogically aligned, can yield significant learning benefits. This insight is particularly relevant for public education systems operating under resource constraints (Trucano and Trucano, 2016).

Policymakers are encouraged to include physical education within broader digital transformation strategies rather than limiting innovation initiatives to cognitively oriented subjects. Such inclusive approaches are consistent with international policy frameworks advocating for equitable and context-sensitive integration of AI in education (Miao and Holmes, 2021).

Ethical considerations are also paramount. The collection and analysis of movement-related data raise concerns related to privacy, consent, and data governance. Clear institutional guidelines and regulatory frameworks are necessary to ensure the responsible use of AI in school settings, in line with ethical principles articulated in human-centered AI frameworks (Floridi et al., 2018).

### 7.4 Directions for future research

Building on the present findings, several avenues for future research can be identified. Longitudinal studies are needed to examine the durability of learning gains and motivational effects associated with AI-supported instruction. Such designs would enable researchers to assess the stability and transfer of motor skills over extended periods, in line with motor learning research emphasizing delayed retention as a key indicator of learning (Schmidt et al., 2018).

Future investigations could also explore hybrid pedagogical models that combine AI-supported tools with established instructional approaches in physical education, such as models-based practice or cooperative learning frameworks. Examining interactions between pedagogical models and AI-supported feedback systems may yield insights into optimal integration strategies across diverse instructional contexts (Casey and Kirk, 2020).

Qualitative and mixed-methods studies would further enrich understanding of how students and teachers perceive and appropriate AI-supported instruction in everyday practice. Such approaches can illuminate the subjective meanings attributed to AI-supported feedback and the pedagogical rationales guiding teachers' use of technological tools, contributing to more contextually grounded design principles (Williamson and Eynon, 2020).

Future studies should build upon the present findings by employing larger and more diverse samples, randomized designs where feasible, objective measures of motor performance, assessor blinding, AI usage analytics, and mixed-methods approaches. Such methodological developments would strengthen causal inference and provide a deeper understanding of how AI-supported instruction influences learning processes across different educational contexts.

### 7.5 Theoretical contributions

From a theoretical standpoint, this study contributes to the literature by empirically integrating perspectives from motor learning theory, self-determination theory, and distributed cognition frameworks to examine AI-supported instruction in physical education. The convergence of performance, motivational, and engagement-related outcomes supports the view that learning processes in embodied contexts are shaped by the interplay of cognitive, affective, and social dimensions (Ennis, 2017).

By conceptualizing AI as a mediating artifact within distributed learning systems, the study advances human-centered approaches to AI in education that foreground the relational and contextual dimensions of technological integration (Salomon, 1998). This theoretical positioning challenges technocentric narratives and reinforces the importance of pedagogical mediation in realizing the educational potential of intelligent technologies (Holmes et al., 2022).

### 7.6 Summary

In summary, while acknowledging methodological constraints, the present study demonstrates that AI-supported instruction can meaningfully enhance motor learning, intrinsic motivation, and student engagement in secondary school physical education when embedded within teacher-mediated pedagogical designs. The implications extend beyond physical education, offering insights relevant to broader discussions on human-centered AI integration in educational practice (Luckin and Holmes, 2016).

Together, the limitations, implications, and future research directions outlined in this section provide a balanced perspective on the contributions and boundaries of the present study. They underscore the need for continued, context-sensitive inquiry into how AI-supported tools can be designed and implemented to support learning in ethically and pedagogically responsible ways.

## 8 Conclusion and recommendations

### 8.1 General conclusion

This study examined the pedagogical impact of integrating AI-supported instructional tools into secondary school physical education within the Tunisian context. By adopting a quasi-experimental design and mobilizing validated instruments, the research provides empirical evidence that AI-supported instruction, when embedded within teacher-mediated pedagogical practices, can significantly enhance motor learning, intrinsic motivation, and student engagement. These findings should be interpreted within the methodological boundaries of the present quasi-experimental study and therefore should not be generalized beyond comparable educational contexts without further empirical evidence.

These findings reinforce the argument that artificial intelligence derives its educational value not from automation alone, but from its alignment with learning theories and pedagogical intent. In the present study, AI-supported tools functioned as mediating artifacts that enriched feedback processes and supported learners' self-regulation without diminishing the embodied, relational, and experiential dimensions of physical education. This human-centered orientation aligns with contemporary perspectives in educational psychology emphasizing the centrality of teacher agency and learner autonomy in technology-enhanced learning environments (Holmes et al., 2022).

Importantly, the study demonstrates that meaningful AI integration is feasible within public secondary schools operating under resource constraints. This challenges deterministic narratives that associate educational innovation with technological sophistication and underscores the importance of pedagogical coherence over technical complexity (Trucano and Trucano, 2016).

### 8.2 Contributions to the field of educational psychology and AI in education

From a scholarly perspective, the study contributes to the literature on AI in education in several ways. First, it extends empirical research beyond cognitively oriented disciplines by focusing on physical education, a practice-based domain in which learning is embodied and socially mediated. This disciplinary shift responds to calls for greater diversity in AI in education research and highlights the relevance of educational psychology frameworks for interpreting learning processes in embodied contexts (Zawacki-Richter et al., 2019).

Second, the study provides empirical evidence from a developing country context, thereby contributing to a more globally inclusive evidence base. By situating the analysis within Tunisian secondary schools, the research offers context-sensitive insights that complement findings derived from technologically advanced educational systems (UNESCO, 2021).

Third, the integration of motor learning theory, self-determination theory, and distributed cognition frameworks offers a coherent theoretical lens for understanding how AI-supported tools influence learning processes in physical education. This interdisciplinary perspective strengthens the conceptual foundations of AI in education research and invites further inquiry across practice-based learning domains (Schmidt and Lee, 2019; Ryan and Deci, 2000). Future research should examine the long-term effects of AI-supported instruction, replicate these findings with larger and more diverse samples, and investigate how different AI applications influence learning across various physical education activities and educational settings.

### 8.3 Practical recommendations for teachers

Based on the findings of this study, several practical recommendations can be formulated for physical education teachers considering the integration of AI-supported tools:

- *Align AI use with pedagogical objectives*. AI-supported tools should be selected and implemented in direct relation to learning goals, particularly those related to motor skill development and formative feedback (Hattie and Timperley, 2007).
- *Maintain active teacher mediation*. Teachers should remain central in interpreting AI-generated feedback and translating it into meaningful guidance for learners, thereby preserving professional judgment and pedagogical coherence (Wise and Shaffer, 2015).
- *Support learner autonomy and reflection*. AI-supported feedback should be framed as a resource for self-assessment and improvement rather than as a mechanism of control, in line with motivational principles articulated in self-determination theory (Ryan and Deci, 2000).
- *Adopt a gradual and reflective integration strategy*. Introducing AI tools incrementally allows both teachers and students to develop shared understandings of their pedagogical value and limitations.

### 8.4 Recommendations for institutions and policymakers

At the institutional and policy levels, the findings suggest several strategic orientations:

- *Include physical education in digital transformation strategies*. Innovation initiatives should recognize PE as a legitimate site for pedagogical experimentation rather than limiting digital integration to academically oriented subjects (Ennis, 2017).
- *Invest in teacher professional development*. Training programs should address not only technical competencies but also pedagogical, ethical, and interpretive dimensions of AI use in education (Holmes et al., 2022).
- *Adopt context-sensitive implementation models*. Policymakers should avoid one-size-fits-all solutions and instead support flexible approaches adapted to local educational realities (Trucano and Trucano, 2016).
- *Ensure ethical governance and data protection*. Clear guidelines on data privacy, consent, and transparency are essential for safeguarding students' rights in AI-supported learning environments (Floridi et al., 2018).

### 8.5 Final reflections

Artificial intelligence is often portrayed as a disruptive force in education. The findings of this study invite a more nuanced interpretation, highlighting that the educational value of AI emerges through its pedagogical mediation and ethical grounding rather than through technological novelty alone. In physical education, a domain centered on movement, interaction, and embodied experience, AI-supported tools can enrich learning processes when they are integrated in ways that respect the human foundations of teaching and learning.

By foregrounding teacher mediation, learner autonomy, and contextual sensitivity, this study contributes to a vision of AI in education that is not only innovative but also pedagogically responsible and psychologically informed. Overall, the present study contributes to the growing body of research on Artificial Intelligence in Education by providing empirical evidence that AI-supported feedback, when implemented within a human-centered pedagogical framework and guided by teacher expertise, can enhance motor learning, intrinsic motivation, and student engagement in secondary school physical education.

## Data Availability

The original contributions presented in the study are included in the article/supplementary material, further inquiries can be directed to the corresponding author.

## References

[B1] Aleven V. McLaughlin E. A. Glenn R. A. Koedinger K. R. (2016). “L'enseignement basé sur les technologies d'apprentissage adaptatif,” in Manuel de recherche sur l'apprentissage et l'enseignement, Vol. 2, eds. R. E. Mayer, and P. Alexander. (New York, NY: Routledge), 522–560.

[B2] Andy F. (2018). Discovering Statistics using IBM SPSS Statistics. London: SAGE Publications.

[B3] Baca A. Kornfeind P. (2006). Rapid feedback systems for elite sports training. IEEE Pervasive Comput. 5, 70–76. doi: 10.1109/MPRV.2006.82

[B4] Baca A. Kornfeind P. (2012). Stability analysis of motion patterns in biathlon shooting. Hum. Mov. Sci. 31, 295–302. doi: 10.1016/j.humov.2010.05.008 20675002

[B5] Bo N. S. W. (2025). OECD digital education outlook 2023: towards an effective education ecosystem. Hung. Educ. Res. J. 15, 284–289. doi: 10.1556/063.2024.00340

[B6] Camomilla V. Bergamini E. Fantozzi S. Vannozzi G. (2018). Trends supporting the in-field use of wearable inertial sensors for sport performance evaluation: a systematic review. Sensors 18:873. doi: 10.3390/s18030873 29543747 PMC5877384

[B7] Casey A. Kirk D. (2020). Models-Based Practice in Physical Education. London: Routledge. doi: 10.4324/9780429319259

[B8] Chen L. Chen P. Lin Z. (2020). Artificial intelligence in education: a review. IEEE Access 8, 75264–75278. doi: 10.1109/ACCESS.2020.2988510

[B9] Cohen J. (1988). Statistical Power Analysis for the Behavioral Sciences. Hillsdale, NJ*:* Lawrence Erlbaum Associates Inc, 19.

[B10] Cohen J. (2013). Statistical Power Analysis for the Behavioral Sciences. London: Routledge. doi: 10.4324/9780203771587

[B11] Cohen L. Manion L. Morrison K. (2018). “Action research,” in Research Methods in Education, eds. L. Cohen, L. Manion, and K. Morrison (London: Routledge), 440–456. doi: 10.4324/9781315456539-22

[B12] Creswell J. W. Creswell J. D. (2014). Research Desing: Qualitative, Quantitative and Mixed Methods Approaches, Vol. 54. Thousand Oaks, CA: Sage Publications.

[B13] Crompton H. Burke D. (2023). Artificial intelligence in higher education: the state of the field. Int. J. Educ. Technol. High. Educ. 20, 1–22. doi: 10.1186/s41239-023-00392-8

[B14] Darwez F. Jeguirim K. Bryant C. R. (2020). Class size and educational achievement in Tunisia: a spatial econometric approach. Région et Développement 52, 83–104.

[B15] Deci E. L. Ryan R. M. (2008). Self-determination theory: a macrotheory of human motivation, development, and health. Can. Psychol. 49:182. doi: 10.1037/a0012801

[B16] Engeström Y. (2001). Expansive learning at work: toward an activity theoretical reconceptualization. J. Educ. Work 14, 133–156. doi: 10.1080/13639080020028747

[B17] Ennis C. D. (2017). Educating students for a lifetime of physical activity: enhancing mindfulness, motivation, and meaning. Res. Q. Exerc. Sport 88, 241–250. doi: 10.1080/02701367.2017.1342495 28742426

[B18] Field A. (2018). Discovering Statistics Using IBM SPSS Statistics (5th ed.). SAGE Publications.

[B19] Floridi L. Cowls J. Beltrametti M. Chatila R. Chazerand P. Dignum V. . (2018). AI4People—an ethical framework for a good AI society: opportunities, risks, principles, and recommendations. Minds Mach. 28, 689–707. doi: 10.1007/s11023-018-9482-5 30930541 PMC6404626

[B20] Fredricks J. A. Blumenfeld P. C. Paris A. H. (2004). School engagement: potential of the concept, state of the evidence. Rev. Educ. Res. 74, 59–109. doi: 10.3102/00346543074001059

[B21] Goodyear V. A. Casey A. Kirk D. (2014). Hiding behind the camera: social learning within the cooperative learning model to engage girls in physical education. Sport Educ. Soc. 19, 712–734. doi: 10.1080/13573322.2012.707124

[B22] Guadagnoli M. A. Lee T. D. (2004). Challenge point: a framework for conceptualizing the effects of various practice conditions in motor learning. J. Mot. Behav. 36, 212–224. doi: 10.3200/JMBR.36.2.212-224 15130871

[B23] Hattie J. Timperley H. (2007). The power of feedback. Rev. Educ. Res. 77, 81–112. doi: 10.3102/003465430298487

[B24] Henrie C. R. Halverson L. R. Graham C. R. (2015). Measuring student engagement in technology-mediated learning: a review. Comput. Educ. 90, 36–53. doi: 10.1016/j.compedu.2015.09.005

[B25] Holmes W. Bialik M. Fadel C. (2019). Artificial Intelligence in Education Promises and Implications for Teaching and Learning. Center for Curriculum Redesign: Center for Curriculum Redesign.

[B26] Holmes W. Porayska-Pomsta K. Holstein K. Sutherland E. Baker T. Shum S. B. . (2022). Ethics of AI in education: towards a community-wide framework. Int. J. Artif. Intell. Educ. 32, 504–526. doi: 10.1007/s40593-021-00239-1

[B27] Kirk D. (2009). Physical Education Futures. London: Routledge. doi: 10.4324/9780203874622

[B28] Koekoek J. Van Hilvoorde I. (2019). Digital Technology in Physical Education. London: Routledge. doi: 10.4324/9780203704011

[B29] Lakens D. (2013). Calculating and reporting effect sizes to facilitate cumulative science: a practical primer for t-tests and ANOVAs. Front. Psychol. 4:863. doi: 10.3389/fpsyg.2013.00863 24324449 PMC3840331

[B30] Luckin R. Holmes W. (2016). Intelligence Unleashed: An Argument for AI in Education. London: Pearson/UCL Knowledge Lab.

[B31] Miao F. Holmes W. (2021). AI and Education: A Guidance for Policymakers. Paris: UNESCO Publishing.

[B32] Mohammadi F. Bahram A. Khalaji H. Ulrich D. A. Ghadiri F. (2019). Evaluation of the psychometric properties of the Persian version of the test of gross motor development−3rd edition. J. Mot. Learn. Dev. 7, 106–121. doi: 10.1123/jmld.2017-0045

[B33] Newell K. M. (1986). “Constraints on the development of coordination,” in Motor Development on Children: Aspects of Coordination and Control, eds. M. G. Wade, and H. T. A. Whiting (Dordrecht: Martinus Nijhoff), 341. doi: 10.1007/978-94-009-4460-2_19

[B34] Ouyang F. Zheng L. Jiao P. (2022). Artificial intelligence in online higher education: a systematic review of empirical research from 2011 to 2020. Educ. Inf. Technol. 27, 7893–7925. doi: 10.1007/s10639-022-10925-9

[B35] Pardo A. Siemens G. (2014). Ethical and privacy principles for learning analytics. Br. J. Educ. Technol. 45, 438–450. doi: 10.1111/bjet.12152

[B36] Reeve J. (2012). “A self-determination theory perspective on student engagement,” in Handbook of Research on Student Engagement, eds. S. L. Christenson, A. L. Reschly, and C. Wylie (Boston, MA: Springer US), 149–172. doi: 10.1007/978-1-4614-2018-7_7

[B37] Roll I. Wylie R. (2016). Evolution and revolution in artificial intelligence in education. Int. J. Artif. Intell. Educ. 26, 582–599. doi: 10.1007/s40593-016-0110-3

[B38] Ryan R. M. Deci E. L. (2000). Intrinsic and extrinsic motivations: classic definitions and new directions. Contemp. Educ. Psychol. 25, 54–67. doi: 10.1006/ceps.1999.1020 10620381

[B39] Saaidia S. Henchiri H. Znazen H. Chaabeni A. Alotaibi A. Alliheibi A. H. . (2026). Basic psychological needs, passion, and well-being at work: evidence from Tunisian physical education teachers. Healthcare 14:1171. doi: 10.3390/healthcare14091171 42121614 PMC13164061

[B40] Salomon G. (1998). Novel constructivist learning environments and novel technologies: Some issues to be concerned with. Learn. Instr. 8(Suppl. 1), 3–12. doi: 10.1016/S0959-4752(98)00007-3

[B41] Salomon G. (ed.). (1997). Distributed Cognitions: Psychological and Educational Considerations. Cambridge: Cambridge University Press.

[B42] Schindler L. A. Burkholder G. J. Morad O. A. Marsh C. (2017). Computer-based technology and student engagement: a critical review of the literature. Int. J. Educ. Technol. High. Educ. 14:25. doi: 10.1186/s41239-017-0063-0

[B43] Schmidt R. A. Lee T. D. (2019). Motor Learning and Performance: From Principles to Application (6th ed.). Human Kinetics.

[B44] Schmidt R. A. Lee T. D. Winstein C. Wulf G. Zelaznik H. N. (2018). Motor Control and Learning: A Behavioral Emphasis. Champaign, IL: Human Kinetics.

[B45] Selwyn N. (2019). Should Robots Replace Teachers? AI and the Future of Education. Hoboken, NJ: John Wiley and Sons.

[B46] Shadish W. R. (2002). Experimental and Quasi-Experimental Designs for Generalized Causal Inference. Belmont, CA: Wadsworth Cengage Learning.

[B47] Sun J. C. Y. Rueda R. (2012). Situational interest, computer self-efficacy and self-regulation: their impact on student engagement in distance education. Br. J. Educ. Technol. 43, 191–204. doi: 10.1111/j.1467-8535.2010.01157.x

[B48] Trucano M. Trucano M. (2016). Saber-ICT Framework Paper for Policy Analysis: Documenting National Educational Technology Policies Around the World and Their Evolution Over Time. Washington, DC: World Bank. doi: 10.1596/26107

[B49] Ulrich D. A. (2019). Test of Gross Motor Development (3rd ed.). PRO-ED.

[B50] UNESCO. (2021). AI and Education: Guidance for Policy-Makers. UNESCO. doi: 10.54675/PCSP7350

[B51] Williamson B. Eynon R. (2020). Historical threads, missing links, and future directions in AI in education. Learn. Media Technol. 45, 223–235. doi: 10.1080/17439884.2020.1798995

[B52] Wise A. F. Shaffer D. W. (2015). Why theory matters more than ever in the age of big data. J. Learn. Anal. 2, 5–13. doi: 10.18608/jla.2015.22.2

[B53] Wulf G. Lewthwaite R. (2016). Optimizing performance through intrinsic motivation and attention for learning: the OPTIMAL theory of motor learning. Psychon. Bull. Rev. 23, 1382–1414. doi: 10.3758/s13423-015-0999-9 26833314

[B54] Zawacki-Richter O. Marín V. I. Bond M. Gouverneur F. (2019). Systematic review of research on artificial intelligence applications in higher education–where are the educators?. Int. J. Educ. Technol. High. Educ. 16, 1–27. doi: 10.1186/s41239-019-0171-0

